# Association of oesophageal radiation dose volume metrics, neutropenia and acute radiation oesophagitis in patients receiving chemoradiotherapy for non-small cell lung cancer

**DOI:** 10.1186/s13014-016-0596-8

**Published:** 2016-02-11

**Authors:** Sarah Everitt, Mary Duffy, Mathias Bressel, Belinda McInnes, Christine Russell, Tim Sevitt, David Ball

**Affiliations:** Division of Radiation Oncology and Cancer Imaging, Peter MacCallum Cancer Centre, Locked Bag 1, A’Beckett St, Melbourne, 8006 Victoria Australia; Division of Nursing, Peter MacCallum Cancer Centre, Locked Bag 1, A’Beckett Street, Melbourne, 8006 Victoria Australia; Centre for Biostatistics and Clinical Trials, Peter MacCallum Cancer Centre, Locked Bag 1, A’Beckett St, Melbourne, Victoria 8006 Australia; Sir Peter MacCallum Department of Oncology, University of Melbourne, Parkville, Victoria 3010 Australia; Department of Medical Imaging & Radiation Sciences, Faculty of Medicine, Nursing & Health Sciences, Monash University, Clayton, 3800 Victoria Australia; Current address: Kent Oncology Centre, Maidstone & Tunbridge Wells NHS Trust, Kent, UK

**Keywords:** Lung cancer, Neutropenia, Oesophagitis, Radiation toxicity, Radiation therapy

## Abstract

**Introduction:**

The relationship between oesophageal radiation dose volume metrics and dysphagia in patients having chemoradiation (CRT) for non-small cell lung cancer (NSCLC) is well established. There is also some evidence that neutropenia is a factor contributing to the severity of oesophagitis. We retrospectively analysed acute radiation oesophagitis (ARO) rates and severity in patients with NSCLC who received concurrent chemotherapy and high dose radiation therapy (CRT). We investigated if there was an association between grade of ARO, neutropenia and radiation dose volume metrics.

**Material and methods:**

Patients with NSCLC having concurrent CRT who had RT dose and toxicity data available were eligible. Exclusion criteria included previous thoracic RT, treatment interruptions and non-standard dose regimens. RT dosimetrics included maximum and mean oesophageal dose, oesophagus dose volume and length data.

**Results:**

Fifty four patients were eligible for analysis. 42 (78 %) patients received 60 Gy. Forty four (81 %) patients received carboplatin based chemotherapy. Forty eight (89 %) patients experienced ARO ≥ grade 1 (95 % CI: 78 % to 95 %). ARO grade was associated with mean dose (r_s_ = 0.27, *p* = 0.049), V20 (r_s_ = 0.31, *p* = 0.024) and whole oesophageal circumference receiving 20 Gy (r_s_ = 0.32 *p* = 0.019). In patients who received these doses, V20 (*n* = 51, r_s_ = 0.36, *p* = 0.011), V35 (*n* = 43, r_s_ = 0.34, *p* = 0.027) and V60 (*n* = 25, r_s_ = 0.59, *P* = 0.002) were associated with RO grade. Eleven of 25 (44 %) patients with ARO ≥ grade 2 also had ≥ grade 2 acute neutropenia compared with 5 of 29 (17 %) patients with RO grade 0 or 1 (*p* = 0.035).

**Conclusion:**

In addition to oesophageal dose-volume metrics, neutropenia may also be a risk factor for higher grades of ARO.

## Introduction

Acute radiation oesophagitis (ARO) is a common toxicity associated with thoracic radiotherapy (RT) when the treated volume includes the mediastinum. The incidence and severity of the resulting dysphagia is related to the dose and volume of oesophagus treated [1, 2]. In patients receiving treatment for lung cancer with curative intent, the administration of concurrent chemotherapy has been shown to improve survival compared with sequential administration [3]. However, concurrent treatment is also associated with increased oesophageal toxicity. This may be the result of a direct radiosensitising effect of the chemotherapy, or it could be an indirect effect of chemotherapy induced neutropenia which results in impaired healing of the oesophageal epithelium. In support of this latter hypothesis, de Ruysscher et al. observed a significant association between the maximal grade of neutropenia and severity of ARO during chemoradiotherapy (CRT) for NSCLC [4].

The primary aim of this retrospective analysis was to assess the rates of acute oesophagitis in a cohort of patients treated at our centre, with a particular focus on dosimetric and haematologic risk factors, to independently verify the findings of de Ruysscher et al.

## Materials and method

Approval was granted by the Peter MacCallum Cancer Centre Institutional Ethics committee to conduct this retrospective study. Criteria for inclusion of patient data in the study included a pathological confirmation of NSCLC and treatment with concurrent chemotherapy and radical or high dose palliative radiotherapy at the Peter MacCallum Cancer Centre (East Melbourne campus). Exclusion criteria included previous thoracic RT, inconsistent radiation dose per fraction and hyper-fractionated radiation schedules. We also excluded patients with RT treatment interruptions ≥ 5 days, as mucosal recovery during the break may have confounded interpretation of the relationship between the oesophageal dose volume metrics and grade of oesophagitis.

Patient characteristics, treatment and toxicity data were extracted from paper and electronic medical records. Clinical data included the use of nasogastric (NG) or percutaneous endoscopic gastrostomy (PEG) tube, systemic steroids, proton pump inhibitors (PPI) and H2 receptor antagonists prior to or during treatment. Patients’ weight loss, smoking status, presence of auto-immune disease, diabetes and/or reflux, and use of analgesia were recorded. Treatment data included total radiation dose, dose per fraction, overall treatment time and chemotherapy schedules. The presence and grade of acute and late oesophagitis, skin toxicity and neutropenia were assessed in accordance with Common Toxicity Criteria for Adverse Events (CTCAE v3.0). Acute toxicities were defined from the first day of treatment to day 90 and late toxicities were defined from day 91 after the start of RT. Toxicity assessments were undertaken by a radiation oncologist at weekly intervals and by a nurse at additional time-points. During treatment neutropenia data were obtained from pre-chemotherapy blood monitoring. The timing of treatment reviews and blood tests varied between patients.

Three-dimensional conformal RT planning was performed on Xio (Computerized Medical Systems CMS, St Louis, MO, USA), using a fast superposition algorithm and delivered with 6MV photons. Planning was performed in accordance with ICRU guidelines, including dose homogeneity within the planning target volume (PTV) of +7 %/-5 % of the prescribed dose. The outer muscular border of the oesophagus was delineated from the cricoid (superior border) to the gastro-oesophageal junction (inferior border) on CT derived images, using soft-tissue window/level settings. Anatomical and dosimetric endpoints included the absolute length (cm) and volume (cm^3^) of oesophagus, maximum (Dmax) and mean (Dmean) oesophageal doses and percentage volume of oesophagus receiving 20 to 60 Gy (Vx, in 5 Gy increments). The percentage length of oesophagus receiving 20 to 60 Gy (in 10 Gy increments) was recorded for dose encompassing the whole oesophageal circumference (LWO) and the partial oesophageal circumference (LPO).

The rate of ARO was described as the percentage of patients in the study who had experienced ARO of at least grade 1. The rate of late oesophagitis and the rate of high grade ARO (grade 3 or 4) were reported in the same manner. All percentages of oesophagitis were reported together with a 95 % confidence interval (95 % CI) based on the binomial distribution. Assessment of potential prognostic factors with respect to ARO was done using Wilcoxon rank sum test and Spearman’s correlation. ARO, acute neutropenia and acute skin reaction were dichotomised as low grade (0 + 1) vs. high grade (2 + 3 + 4). The association of ARO with acute neutropenia and acute skin reaction was examined using Barnard’s test. All *p*-values are 2-sided without adjustment for multiplicity. All statistical analyses were performed in R (R Development Core Team (2012). R: A language and environment for statistical computing. R Foundation for Statistical Computing, Vienna, Austria. ISBN 3-900051-07-0, URL http://www.R-project.org/).

## Results

Eighty four patients met the eligibility criteria for inclusion in this study. The archived radiotherapy planning data of 30 patients were irretrievable; therefore these patients were excluded from the analysis. Characteristics of the remaining 54 patients are presented in Table 1.

**Table 1:** Patient characteristics and clinical factors

Variable	Category	Count	%
Gender	Female	14	25.9
	Male	40	74.1
T Stage	1	4	7.4
	2	22	40.7
	3	13	24.1
	4	13	24.1
	X	2	3.7
N Stage	0	13	24.1
	1	8	14.8
	2	26	48.1
	3	6	11.1
	X	1	1.9
M Stage	0	46	85.2
	1	5	9.3
	X	3	5.6
Smoking	Current smoker	9	16.7
	Ex-smoker (<6 months)	10	18.5
	Ex-smoker (> =6 months)	32	59.3
	Never smoked	3	5.6
Autoimmune disease	No	52	96.3
	Yes	2	3.7
Diabetes	No	46	85.2
	Yes	8	14.8
Reflux oesophagitis	No	42	77.8
	Yes	12	22.2

Table 2 shows the treatment details for CRT and any prescribed other therapies including steroids and analgesia. 42 (78 %) patients received CRT with a radical dose of 60 Gy. The remaining 12 (22 %) patients received high palliative doses ranging between 30 and 50 Gy, details of which have been published previously [5]. No patient experienced a treatment break. The median change in weight from the start to the end of RT was -1Kg, ranging from −8.5Kg to 3.5Kg.

**Table 2:** Treatment details

Variable	Category	Count	%
RT dose received	<60Gy	12	22.2
	60Gy	42	77.8
RT type	High dose palliative	10	18.5
	Radical	44	81.5
RT breaks	No	54	100
	Yes	0	0
Chemo regimen	Carboplatin + paclitaxel + gefitinib	4	7.4
	Carboplatin + paclitaxel weekly	20	37
	Carboplatin weekly	3	5.6
	Carboplatin weeks 1 and 6	17	31.5
	Gemcitabine weekly	8	14.8
	Vinorelbine + Cisplatin weekly	2	3.7
Neoadjuvant chemo	No	52	96.3
	Yes^a^	2	3.7
NGPEG pre CRT	No	54	100
	Yes	0	0
NGPEG during CRT	No	53	98.1
	Yes	1	1.9
Steroids pretreatment	No	49	90.7
	Yes	5	9.3
Steroids during	No	27	50
	Yes	27	50
PPI pre	No	45	83.3
	Yes	9	16.7
PPI during	No	34	63
	Yes	20	37
H2Antag pre CRT	No	52	96.3
	Yes	2	3.7
H2Antag during CRT	No	46	85.2
	Yes	8	14.8
Local analgesia	No	17	31.5
	Yes	37	68.5
Type local analgesia	Aluminium hydroxide and oxethazaine (Mucaine)	12	32.4
	Lignocaine viscous and Mucaine	22	59.5
	Lignocaine viscous only	3	8.1
Systemic analgesia	No	18	33.3
	Yes	36	66.7
Type sys analgesia	non-opioid	8	22.2
	strong opioid	21	58.3
	weak opioid	7	19.4

^a^Of the 2 patients who received neoadjuvant chemotherapy, 1 received Carboplatin + Paclitaxel weekly and 1 received Carboplatin + Gemcitabine

*NGPEG* Nasogastric tube or PEG

*PPI* proton pump inhibitor

*H2antag* Histamine H2 receptor antagonist

The incidence and grade of acute and late oesophageal toxicities are presented in Table 3. Of the 54 patients included in the trial, 48 (89 %) had ARO of at least grade 1 (95 % CI: 78 % to 95 %) and 5 (9 %) patients had late oesophagitis of at least grade 1 (95 % CI: 4 % to 20 %). Of the 29 patients with ARO grade ≤ 1, 5 (17 %) experienced high grade acute neutropenia (grade ≥ 2). In contrast, of the 25 patients with high grade ARO, 11 (44 %) experienced high grade acute neutropenia (*p* = 0.035). The maximum grade of oesophagitis was not significantly associated with the maximum grade of acute skin reaction (*p* = 0.529). There was not a statistically significant association between grade of ARO and sex (*p* = 0.722) or dose, when dichotomised to <60 Gy and 60 Gy (*p* = 0.401).

**Table 3:** Description of toxicities

Variable	Category	Count	% [95 % CI]
Grade of acute oesophagitis	0	6	11.1 [5.2 - 22.2]
	1	23	42.6 [30.3 - 55.8]
	2	17	31.5 [20.7 - 44.7]
	3	8	14.8 [7.7 - 26.6]
Late oesophagitis recorded	No	25	46.3 [33.7 - 59.4]
	Yes	29	53.7 [40.6 - 66.3]
Grade of late oesophagitis	0	24	82.8 [65.5 - 92.4]
	1	2	6.9 [1.9 - 22.0]
	2	3	10.3 [3.6 - 26.4]
Reason no late oesophagitis	Disease progression	6	25.0 [12.0 - 44.9]
	New second primary	1	4.2 [0.2 - 20.2]
	Not documented in clinical record	9	37.5 [21.2 - 57.3]
	Patient died before first assessment	3	12.5 [4.3 - 31.0]
	Patient had surgery	1	4.2 [0.2 - 20.2]
	Patient lost to F/U	4	16.7 [6.7 - 35.9]

The mean (SD) oesophageal length was 22.4 cm (2.2) and oesophageal volume was 86.9 cm^3^ (16.6). The mean (SD) RT field length was 14.2 cm (3.9). Table 4 illustrates details of the RT dosimetric data and the association of oesophageal dose with ARO grade for all patients and the subset of patients where the oesophagus received the pre-specified dosimetric endpoint. For all patients, the mean (SD) oesophageal Dmax was 50.2 Gy (18) and the mean (SD) oesophageal Dmean was 20.8 Gy (10.8).

**Table 4:** Radiation therapy dosimetric data and the relationship of oesophageal dose with grade of acute oesophagitis

	All patients (*n* = 54)				Only patients receiving pre-specified dose to the oesophagus		
Variable	Mean	sd	Spearman correlation	*p*-value	*n*	Spearman correlation	*p*-value
Dmax (Gy)	50.2	18.0	0.143	0.302	-	-	-
Dmean (Gy)	20.8	10.8	0.269	**0.049**	-	-	-
VO20	43.9	23.1	0.306	**0.024**	51	0.355	**0.011**
VO25	40.8	22.1	0.297	**0.029**	50	0.303	**0.033**
VO30	34.8	23	0.238	0.083	49	0.269	0.062
VO35	30.8	24.4	0.201	0.146	43	0.337	**0.027**
VO40	26.9	23.9	0.162	0.243	41	0.302	0.055
VO45	23.8	23	0.163	0.24	38	0.310	0.058
VO50	21.1	21.1	0.152	0.273	38	0.289	0.078
VO55	17.1	19.1	0.124	0.371	37	0.290	0.081
VO60	6.9	10.9	0.104	0.455	25	0.591	**0.002**
LPO20	48.9	21	0.205	0.136	51	0.240	0.090
LPO30	42.2	21.4	0.134	0.336	50	0.107	0.458
LPO40	34.2	24.7	0.106	0.448	41	0.206	0.196
LPO50	29.9	24	0.101	0.469	38	0.189	0.255
LPO60	12.5	16.1	0.048	0.728	27	0.428	**0.026**
LWO20	29.8	22.7	0.319	**0.019**	43	0.185	0.236
LWO30	20.5	20.4	0.250	0.068	36	0.148	0.389
LWO40	14.4	18.6	0.231	0.093	30	0.248	0.186
LWO50	8.6	14.1	0.210	0.128	20	0.214	0.364
LWO60	1	3.8	0.139	0.316	6	0.147	0.781

*VOx* Percentage volume of oesophagus receiving x dose (%), *LPOx* Percentage length of partial oesophagus receiving x dose (%), *LWOx* Percentage length of whole oesophagus receiving x dose (%). Values in bold type indicate statistical significance, *p* < 0.05

When analysing the association between ARO grade and Vx parameters, there was a statistically significant correlation between the grade of ARO and percentage of oesophagus treated at 20 Gy for the whole patient cohort (*n* = 54, r_s_ = 0.306, *p* = 0.024) and the subset of patients with oesophagus volumes treated to 20 Gy (*n* = 51, r_s_ = 0.355, *p* = 0.011). The correlation between the grade of ARO and percentage of oesophagus treated at 60 Gy was not statistically significant for the whole cohort (*n* = 54, r_s_ = 0.104, *p* = 0.455) but highly significant for the subset of patients receiving 60 Gy to the oesophagus (*n* = 25, r_s_ = 0.591, *p* = 0.002). As shown in Table 4, the percentage of oesophagus receiving at least 25 Gy (*n* = 50, r_s_ = 0.303, *p* = 0.033), 35 Gy (*n* = 43, r_s_ = 0.337, *p* = 0.027) were also statistically significant in the subset of patients receiving 25 Gy and 35 Gy to the oesophagus, respectively.

**Fig. 1:**
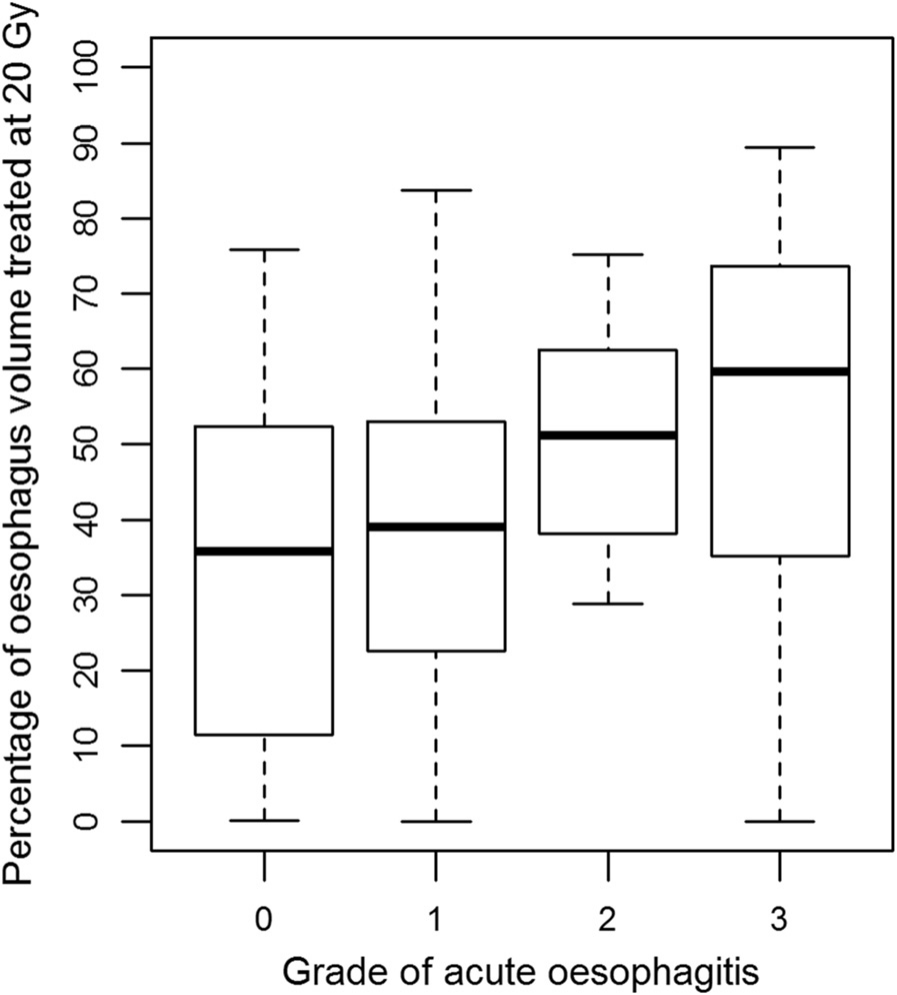
Box-and-whisker plot showing the percentage of oesophagus volume treated at 20Gy according to grade of acute oesophagitis

**Fig. 2:**
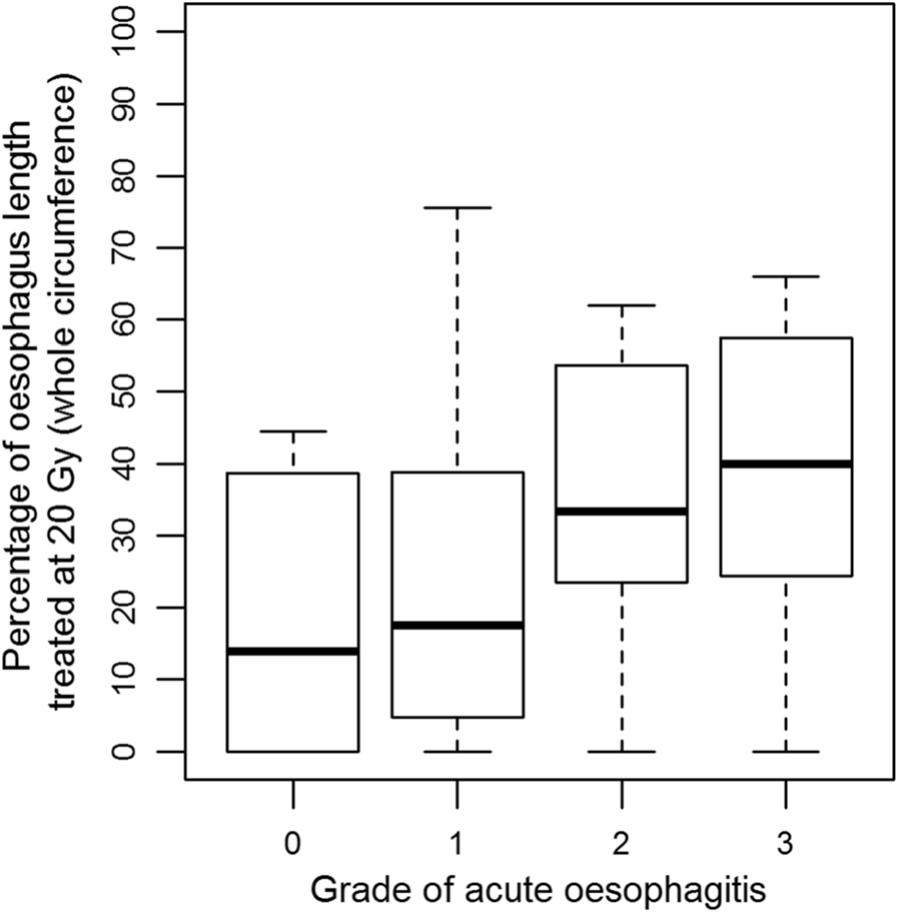
Box-and-whisker plot showing the percentage of oesophagus length treated at 20Gy (whole circumference) according to grade of acute oesophagitis

The analysis of association between ARO grade and irradiated length showed a statistically significant correlation in patients receiving 60 Gy to the partial oesophageal circumference (*n* = 27, r = 0.428, *p* = 0.026) but not in patients where the whole oesophageal circumference was irradiated to 60 Gy (*n* = 6, r = 0.147, *p* = 0.781). For the whole patient cohort, only the whole oesophageal circumference treated to 20 Gy was statistically significantly associated with acute oesophagitis grade (r = 0.319, *p* = 0.019). Fig. 1 and Fig. 2 illustrate the relationship of grade of acute oesophagitis with volume and length receiving 20 Gy, respectively.

The relationship of neutropenia and oesophagitis was assessed on all patients and also separately in the subsets of patients with >50 % of oesophagus volume receiving 20Gy and ≤50 % of oesophagus volume receiving 20Gy and the results presented in Table 5. Higher grades of neutropenia were associated with higher grades of oesophagitis when assessed in the whole cohort. Despite not achieving statistical significance, the results from both volume subsets were consistent with the result for the whole cohort, probably a reflection of the small number of patients in the subsets. If however the relationship between oesophagitis and neutropenia is tested adjusting for percentage of oesophagus volume receiving 20Gy (as a continuous variable), neutropenia was associated with oesophagitis (OR = 3.82 95 % CI [1.06 – 15.79], *p* = 0.048).

**Table 5:** Grade of oesophagitis and grade of neutropenia according to percentage of oesophagus receiving 20 Gy

% of oesophagus volume receiving 20Gy	Acute neutropenia grade	Acute oesophagitis grade		*p*-value
		0 + 1	2 + 3 + 4	
50 % or less	0 + 1	17 (85 %)	6 (55 %)	0.071
	2 + 3 + 4	3 (15 %)	5 (45 %)	
>50 %	0 + 1	7 (78 %)	8 (57 %)	0.400
	2 + 3 + 4	2 (22 %)	6 (43 %)	
Overall	0 + 1	24 (83 %)	14 (56 %)	0.035
	2 + 3 + 4	5 (17 %)	11 (44 %)	

## Discussion

ARO is a common, painful and debilitating toxicity associated with high dose CRT for locally advanced NSCLC, and mitigation of the risk of high grade ARO should be a priority of thoracic radiation oncology research. Oesophageal dose and volume are well recognised risk factors [1, 2], and our data are consistent with the literature in this regard. All of our patients received concurrent chemotherapy, also a recognised risk factor, although it is not known whether as a result of direct radiosensitisation of the oesophageal mucosa, or indirectly through chemotherapy induced neutropenia, or a combination of both. We have shown a significant association between grade of ARO and grade of neutropenia, supporting the previous findings of de Ruysscher et al. in favour of an indirect effect [4]. The implications are that existing models predicting severity of ARO based purely on oesophageal dose volume metrics [1] are oversimplified and may need refinement by adjustment for the effect of neutropenia.

It is not surprising that there should be a relationship between grade of ARO and neutropenia. Chemotherapy and RT are known to reduce the integrity of the oesophageal mucosal epithelium, thereby damaging the first line of defence against pathogens [6]. Healing of the mucosa will be delayed if the inflammatory response is impaired due to a lack of neutrophils. Indeed, prophylactic G-CSF has been investigated for its ability to reduce mucositis in patients having radiotherapy for head and neck cancer. In one randomised trial, G-CSF was associated with a non-significant reduction in mucositis, but also a reduction in local cancer control [7].

In de Ruysscher’s study, neutropenia was only observed in patients who received CRT and it was the most significant parameter for predicting ARO [4]. Although our patient numbers were limited, they are consistent with the findings of de Ruysscher et al. and we are not aware of any other reports implicating neutropenia as a contributory factor to the grade of ARO.

Several dosimetric endpoints in our study were statistically associated with ARO. These included the mean oesophageal dose (*p* = 0.049), consistent with some previous studies [1]. Low doses to large oesophageal volumes were statistically significantly associated with high oesophageal toxicity in our cohort of patients receiving CRT, including the volume receiving 20Gy (*p* = 0.026) and whole oesophageal circumference receiving 20Gy (*p* = 0.019). In the subset analysis of patients treated to given doses, the oesophagus V60 was also associated with oesophagitis (r_s_ = 0.591, *p* = 0.002). Although this finding represents a small subset of 25 patients, it is consistent with other literature, including Bradley et al., who reported the association between oesophagitis and V60 in a study of 166 patients [8]. Similar to our own findings, Takeda et al. [9] and Belderbos et al. [10] concluded that the most significant dosimetric parameter was the volume of oesophagus receiving at least 35Gy (*p* < 0.001). These data were based on the incidence of ≥ Grade 2 oesophagitis, whereas our own data are for ≥ Grade 1 toxicity.

The proximity of primary lung cancers and mediastinal nodes to the oesophagus in many CRT candidates means that inclusion of the oesophagus within the treated volume is often unavoidable, even with IMRT techniques. The opportunities for reducing oesophageal dose and volume are therefore limited. Our results suggest that an alternative strategy worth investigating may be the use of concurrent chemotherapy regimens which are less likely to cause myelosuppression but without loss of therapeutic benefit.

## Conclusions

This study suggests that in patients receiving concurrent chemoradiotherapy for NSCLC risk factors for radiation oesophagitis include not only oesophageal dose and volume, but also neutropenia. The determination of oesophageal dose volume constraints may not be reliable unless this is taken into account. It may be possible to reduce the risk of high grade oesophagitis by choosing a concurrent chemoradiotherapy regimen with a low likelihood of myelosuppression.
